# An operationalization framework for lifecycle health technology assessment: a Health Technology Assessment International Global Policy Forum Task Force report

**DOI:** 10.1017/S0266462324000199

**Published:** 2024-05-16

**Authors:** Franz B. Pichler, Meindert Boysen, Nicole Mittmann, Ramiro Gilardino, Andrew Bruce, Kenneth Bond, Rick A. Vreman, Nathalie Largeron, Judit Banhazi, Daniel A. Ollendorf, Mohit Jain, Sheela Upadhyaya, Wim G. Goettsch

**Affiliations:** 1 Confluence Health Consulting, Sydney, NSW, Australia; 2 National Institute for Health and Care Excellence (NICE), London, UK; 3 Canadian Agency for Drugs and Technologies in Healthcare, Ottawa, ON, Canada; 4 MSD, Zurich, Switzerland; 5 Amgen, Sydney, NSW, Australia; 6 Institute of Health Economics, Edmonton, AB, Canada; 7 Roche, Utrecht, Netherlands; 8 Sanofi, Paris, France; 9 Menarini, Zurich, Switzerland; 10 Tufts Medical Center, Institute for Clinical Research and Health Policy Studies, Boston, MA, USA; 11 BioMarin, London, UK; 12 Life Sciences Consultant, London, UK; 13 Zorginstituut Nederland (ZIN), Utrecht, Netherlands

**Keywords:** Operationalization, lifecycle, checklist, practical, guidance, examples, sequencing, criteria

## Abstract

Operationalization guidance is needed to support health technology assessment (HTA) bodies considering implementing lifecycle HTA (LC-HTA) approaches. The 2022 Health Technology Assessment International (HTAi) Global Policy Forum (GPF) established a Task Force to develop a position paper on LC-HTA. In its first paper, the Task Force established a definition and framework for LC-HTA in order to tailor it to specific decision problems. This second paper focused on the provision of practical operational guidance to implement LC-HTA. Detailed descriptions of the three LC-HTA operational steps are provided (defining the decision problem, sequencing of HTA activities, and developing optimization criteria) and accompanied by worked examples and an operationalization checklist with 20 different questions for HTA bodies to consider when developing an LC-HTA approach. The questions were designed to be applicable across different types of HTA and scenarios, and require adaptation to local jurisdictions, remits, and context.

## Introduction

A multistakeholder Task Force was developed as an output of the 2022 Health Technology Assessment International (HTAi) Global Policy Forum on the topic of lifecycle (LC) approaches to HTA (1). The Task Force developed two companion papers describing and addressing the challenges associated with lifecycle HTA (LC-HTA). The first paper (2) described the strategic reasons why LC-HTA would be of value to health technology assessment (HTA) bodies and presented a definition for LC-HTA. Four scenarios were identified where an LC-HTA approach might provide added values, which were (i) where the initial information about the technology is limited, (ii) where an individual technology may be modified over its lifecycle, (iii) where a learning curve related to utilizing a technology in practice changes its outcomes and (iv) where the health service context impacts or is changed by the technology. These diverse scenarios led to the conclusion that LC-HTA approaches require tailoring to the decision problem. A Framework was developed to describe the three key components of an LC-HTA process: (i) defining the decision problem, (ii) sequencing of HTA activities, and (iii) developing optimization criteria.

The focus of this companion paper is to describe and discuss operational considerations for HTA bodies that are considering developing LC-HTA approaches. The first section of the paper provides operational guidance using the LC-HTA framework developed in the first paper, including a high-level checklist and descriptions of each of the framework steps from an operational perspective. Two examples, accelerated regulatory approval and incremental modification of technologies are used to illustrate how to develop an LC-HTA approach using the LC-HTA framework. Following this, we discuss four key topics that the Task Force believes HTA bodies should consider when developing LC-HTA processes. We recognize that HTA bodies may implement LC-HTA processes for all, some, or just one of the potential scenarios that the Task Force considers suitable for LC-HTA.

Goals of this paper are

1. to provide operational guidance on the three key components of an LC-HTA process accompanied by worked examples and a checklist, and
2. to discuss four critical operationalization considerations.

Development of an operationalization checklist:

The Task Force developed an operationalization checklist (Table 1) for the LC-HTA Framework to help provide practical guidance to HTA bodies that are considering utilizing LC-HTA approaches to address a decision problem. The checklist was developed through a discussion of the literature, TF member experience, and outreach to operationalization experts within NICE and CADTH. The operationalization checklist is intended to be a high-level summary of important considerations within each of the three steps of the Framework. The intention is that HTA bodies can apply this checklist during the process of developing an LC-HTA approach relevant to any of the potential LC-HTA scenarios. Each step of the Framework is described in further detail below.

**Table 1.** Steps to tailor a life-cycle HTA approach

High–level checklist for developing an LC–HTA approach		
1	Define the decision problem	
	The development of a clear definition of the decision problem will be used to identify where and why in the technology lifecycle to apply LC–HTA and for what outcome. A key element is to identify if resolving the decision problem through the additional activity will be sufficiently meaningful to justify the resources spent.	
		Has an issue been identified by stakeholders or the HTA body that is likely to cause a clear challenge to existing HTA processes?
		Is this issue a shared priority to address for the HTA body or in the views of key stakeholders?
		Has the issue been articulated as a decision problem statement that expresses the goal of addressing the issue?
		Would the outcome of an LC–HTA approach be reasonably likely to meaningfully address this problem in order to achieve the defined goal?
		Have relevant stakeholders been consulted with respect to the decision problem and the meaningfulness of the potential outcome of an LC–HTA approach?
		Does the value to the HTA body and key stakeholders in addressing this decision problem using LC–HTA exceed the expected resource costs?
		Have alternative approaches to LC–HTA been considered to solve the same problem?
2	Sequencing of HTA activities	
	To resolve the decision problem, determine which HTA activities are required and how they should be connected. An important additional aspect of this step will be in deciding which stakeholders to involve and for what steps.	
		Has the scope (timeframe across the LC) that frames the decision problem been defined?
		Have all HTA activities required to address the decision problem been identified, the reason for their inclusion articulated, and their roles defined with respect to addressing the problem?
		Are all the HTA activities required available in the jurisdiction and fit for purpose? Do any of these activities need to be revised? If an activity is not available within the jurisdiction, is there an option to develop the activity or to partner with a provider of that activity from outside the jurisdiction?
		Have the HTA activities been placed into a clear sequence across the LC that specifies how the different HTA activities are connected?
		Have all relevant stakeholders required for each part of the LC–HTA sequence been identified and engaged with to determine when, how, and why to include them?
		Have the responsibilities of each relevant stakeholder across the sequence been determined, including a communication plan?
		Has there been a feasibility assessment, including the identification of funding/resourcing requirements, across the full sequence of activities?
		Can the proposed sequence of activities be implemented under the existing remit of the HTA body, or are legal/regulatory actions required?
3	Optimization criteria	
	Developing clear criteria or guidelines to determine (i) whether a given technology is eligible for an LC–HTA approach and (ii) when a step in the LC–HTA approach should be triggered to ensure activities are undertaken only when worthwhile.	
		Have clear eligibility criteria been developed to ensure only the intended technologies relevant to the decision problem are included in the process?
		Have relevant stakeholders been consulted, do they understand, and are they aligned on the eligibility criteria?
		Have trigger mechanisms been developed that are placed before key steps in the sequence of HTA activities, especially resource–intensive steps
		Have relevant stakeholders been consulted in developing the trigger mechanisms to ensure alignment and avoid unintended consequences?
		Have the responsibilities for determining identification, monitoring, notification, and actions resulting from trigger criteria been determined?

This table is intended to support HTA bodies in their operationalization of LC-HTA. The table is based on the LC-HTA Framework (2) and provides high-level questions that we believe are important for HTA bodies to consider when developing an LC-HTA approach. The questions are designed to be appliable across different types of HTA and to be suitable for tailoring to the range scenarios (2) where might be applicable. Therefore, individual HTA bodies will need to adapt this checklist to their specific jurisdiction, remit and context.

## Define the decision problem

Articulating a decision problem will guide the scope of HTA activities required to address that problem and enable HTA bodies to use this information to consider the opportunity cost of undertaking this additional work. It is important to determine if an LC-HTA approach would add significant value to addressing the decision problem compared with the alternatives.

Considering that the value of addressing the decision problem and consequent actions, as a result, will differ by stakeholder (3), it is important to ensure stakeholder participation to inform the identification, defining, and prioritization of decision problems. It will be important to communicate such prioritization to stakeholders clearly and transparently (4).

- Example: HTA response to Accelerated Regulatory Approval
  The decision problem for an HTA of a technology with an accelerated regulatory approval relates to how to enable prompt patient access to technologies that potentially can address high unmet needs when the initial evidence base is lower than standard levels for acceptance within HTA. Key questions that impact decision risk are (i) the consequences of the initial decision (e.g., clinical, financial, and so) and (ii) whether the plans for future evidence development will likely address critical evidentiary deficiencies. Utilizing an LC-HTA approach might facilitate foresight on anticipated risks and enable management of the uncertainty associated with the initial evidence base as well as encourage the development of future evidence that addresses HTA concerns.
- Example: Incremental modification of technologies
  The decision problem is when and how HTA bodies should address changes to a technology that impact key elements of the technology’s value. When a change occurs to an existing technology, four key questions arise to determine if a reassessment would be informative: (i) whether the change is sufficiently meaningful to warrant a new review, (ii) at what point should the review be triggered, (iii) the range of evidence required, and (iv) the source(s) of the evidence. LC-HTA is well suited to enabling HTA bodies to address the four underlying questions related to the challenge of incremental modification of technologies.

## Sequencing of HTA activities

Following the definition of a decision problem, it will be important to apply an LC-HTA process that addresses the decision problem in a focused way. As the resource and burden of the additional HTA activities associated with LC-HTA will impact multiple stakeholders (1), a well-articulated scope that frames the decision problem and intended outputs of the activities will be necessary for buy-in. This may be especially important when the LC-HTA process’s success depends on stakeholders not directly linked to the HTA body (e.g., clinicians involved in collecting evidence). The scope will define the sequence and intensity of HTA activities required to address the decision problem (1). It is important to note that this does not require a unique sequence for every unique technology. Additional resource use could be minimized by utilizing or adapting existing, well-established HTA activities rather than designing *de novo* HTA activities. It may also be possible to find efficiency within HTA activities, for example, by preparing assessment models in anticipation of future changes to the evidence base (5–7).

- Example: HTA response to accelerated regulatory approval
  The potential LC-HTA can commence from the time when a technology enters into regulatory discussions concerning accelerated approval pathways and may extend through a post-launch HTA reassessment (HTR) of the completed confirmatory studies and beyond. We envisage a multi-step LC-HTA process (Table 2), including horizon scanning, scientific advice, initial HTA review, post-authorization evidence development, and HTR. There are likely to be differences in which activities can be included within the LC-HTA process depending on the HTA body’s jurisdiction, resourcing, and available HTA-related activities.
- Example: Incremental modification of technologies
  As the decision problem relates to changes in the safety, effectiveness, or utility of a technology following market access, the scope of the LC-HTA process will likely begin at the time of the first HTA appraisal. We envisage a process that begins by defining what constitutes change sufficient to warrant further assessment, evidence collection, notification and then Health Technology Reassessment (HTR) (Table 3). The IDEAL framework is a structured approach that might be well suited to the process of defining what study outcome measures are relevant, designing further evidence generation requirements, and has the potential to provide proactive R&D guidance towards health system needs (8).

**Table 2.** LC-HTA applied to accelerated regulatory approval

Framework	LC–HTA for accelerated regulatory approval
Context	Accelerated regulatory approval refers to pharmaceuticals and devices that have received expedited marketing authorization based on promising early evidence (e.g., Phase II, single–arm studies); however, this results in HTA–related uncertainty concerning the value of the technology and may also result in an increased decision risk at the time of initial assessment.
The decision problem	How to enable patient access to products potentially addressing high unmet needs when the initial evidence base is lower than standard levels for acceptance within HTA.
Sequencing of HTA activities (*note: steps 1–2 may only be relevant if the HTA body is in the same jurisdiction as the regulator*).	Proactive horizon scanning to identify products entering into regulatory discussions about accelerated regulatory approval to help ensure timely HTA participation in discussions relating to the initial evidence base and confirmatory studies, likely in the form of information provided by the technology developer. Tripartite scientific advice with the regulator intended to increase HTA relevance of the confirmatory studies. HTA advice with the technology developer concerning HTA–specific post–launch studies, especially to address uncertainties that are not likely to be addressed through confirmatory pre–approval studies (e.g., comparative evidence). The initial HTA can confirm or establish HTA–related studies on reducing evidentiary uncertainty and define when a future reassessment will occur. Potentially a conditional reimbursement mechanism or managed entry agreement could be utilized. Following completion of the confirmatory studies and/or HTA–specific studies, the HTA would reassess the technology.
Optimization criteria	Two optimization criteria are proposed; Eligibility into the scheme will be limited to products that meet the regulatory criteria, which can vary between regulatory authorities. The HTA body may wish to consider additional eligibility considerations relevant to their remit and the local health system. It will be important to establish trigger criteria during the post–launch evidence collection phase to determine when it will be optimal to conduct an HTA reassessment, and operationalize the monitoring of the fulfillment of these criteria.

We utilized the LC-HTA Framework to show a hypothetical high-level design for an approach to addressing technologies with an accelerated regulatory approval.

**Table 3.** LC-HTA applied to incremental innovation

Framework	LC–HTA for incremental modification of technologies
Context	Some technologies have the potential to be modified, sometimes rapidly, and as a consequence, can change their effectiveness and safety profiles. Such incremental modification is commonly associated with medical devices (22) but is also observed in genomic diagnostics, digital therapeutics, and improvements to existing medicines (e.g., slow–release formulations).
The decision problem	The decision problem is when and how to address the changes to the technology that impact key elements of the technology’s value.
Sequencing of HTA activities	At the time of an initial HTA appraisal, eligible technologies would be subject to an advisory meeting where the HTA body, the technology developer and relevant stakeholders would define a set of Minimal Trigger Thresholds (MTTs) representing both negative and positive boundaries for each key outcome of interest that would be considered sufficiently meaningful to trigger a notification. Once on the market, if a technology is modified and a change to the outcome exceeds either the negative or positive MTT, then a notification mechanism is triggered. A “targeted’ HTR would assess the impact of the change to the technology, focused on the key outcome(s) that are intentionally or unintentionally impacted by the change. Unaffected aspects would not be reassessed to keep the review as efficient as possible.
Optimization criteria	Eligible technologies would have anticipated, or planned, changes that are intended to impact safety, effectiveness and/or utility to an extent where the impact of such changes would be sufficiently meaningful to warrant a new review. Minimal Trigger Thresholds (MTTs) would be defined. The MTTs would represent both negative and positive boundaries for each key outcome of interest that would be considered sufficiently meaningful to trigger a notification. No action is taken if a technology is modified and a key outcome changes but is less than the predefined trigger threshold. If a change to the outcome exceeds either the negative or positive MTT, then a notification mechanism is triggered.

We utilized the LC-HTA Framework to show a hypothetical high-level design for an approach to addressing technologies subject to incremental modification.

## 3. Optimization criteria

Following the development of the sequence of HTA activities required to address a decision problem, it will be important to establish a set of criteria to ensure the process proceeds efficiently and without undue delay. Where an LC-HTA process has been developed for use with multiple technologies, then eligibility criteria can be defined to restrict the selection of technologies that enter the LC-HTA process to ensure the efficient allocation of resources. Within the LC-HTA sequence of HTA activities, different forms of criteria could be applied, such as qualification criteria to enter into a process step, contractual agreements that define required evidence generation (7), defining endpoints with minimally important differences (5), or pre-specified trigger points (6). Ultimately, such criteria aim to ensure that a step in the LC-HTA process is activated only when conducting that step would be meaningful.

In general, the establishment of technology-specific trigger criteria will require discussion with key stakeholders relevant to the determination of what criteria are relevant, when and what evidence can be collected, and who will collect it. It will be necessary to implement a mechanism to determine when a step should be triggered, for example, how to monitor evidence availability. From a stakeholder perspective, the process for determining when certain HTA activities are worthwhile and when they are should be transparent.

- Example: HTA response to accelerated regulatory approval
  We envisage two forms of trigger criteria that could be applied to technologies with accelerated regulatory approvals. Eligibility into an LC-HTA process will be limited to products that have met the regulatory criteria required for accelerated regulatory approval. The HTA body may wish to consider additional eligibility criteria relevant to their remit and the local health system. Second, it will be important to establish trigger criteria and a monitoring process for the fulfillment of these criteria for the post-launch evidence-collection phase of the LC-HTA to determine when to commence an HTA reassessment.
- Example: Incremental modification of technologies
  For technologies that might be subject to incremental changes after their initial HTA review, we propose a multistakeholder discussion during the HTA appraisal of the technology in order to establish technology-specific trigger criteria (Table 2). We anticipate that the nature of incremental modifications of technologies might require a greater level of specific discussion than for other LC-HTA scenarios. The result of the discussion would be the definition of a set of minimal trigger thresholds (MTTs) representing both negative and positive boundaries for each key outcome of interest that would be considered sufficiently meaningful to trigger a notification. The purpose of such criteria would be to minimize resource expenditure by triggering a reassessment only when the incremental innovation results in a change sufficient to significantly impact the findings of the previous review.

## Four key topics to aid considerations regarding LC-HTA operationalization

The TF identified four topics that it considered important for HTA bodies to consider in the process of operationalizing an LC-HTA approach.

1. Using LC-HTA approaches to encourage robust evidence development,
2. How to use LC-HTA to inform decision-making across the lifecycle,
3. Effective implementation of LC-HTA into the health ecosystem,
4. Challenges for LC-HTA approaches.

## Using LC-HTA approaches to encourage robust evidence development

The LC-HTA decision problem will define whether the focus of evidence development for HTA purposes should be in the pre-license, post-license, or post-launch phase of the lifecycle. HTA bodies need to collaborate with key health system stakeholders, from patients, industry, and researchers to regulatory and health providers, in order to ensure efficient data generation that is focused on priority questions for decision-making (1, 7).

### Early HTA-regulatory advice

The evidence base of a new technology is typically dependent on the technology developer’s global development plans. Tripartite regulatory, HTA, and technology developer advice meetings are a well-established process to identify uncertainties of concern for downstream stakeholders and discuss their inclusion in the evidence development (9). Such advice has influenced development planning (10). In the post-licensing context, there can be continued evidence generation “PLEG” (11) related to regulatory requirements (e.g., pharmacovigilance, confirmatory trials) or to meet the needs of other stakeholders. Coordination between regulatory and HTA bodies in relation to post-licensing regulatory trials has often been confined to interagency information sharing (12), and such regulatory studies often do not address the key concerns of HTA, such as relative effectiveness (9). There are also opportunities for efficiency in evidence development where HTA and regulatory bodies can pre-align on the type of data being generated and analysis methodologies. While there is a discussion about the desirability of improving regulatory and HTA alignment in trial design (1,9,12,13), the difference in remits between these agencies means that evidence gaps will remain (9,13) and may require complementary “HTA-specific” evidence development depending on the decision problem.

### Early HTA advice

HTA advice is an established activity that enables dialogue between a technology developer and one or more HTA bodies in relation to evidence development. Early HTA advice that occurs prior to the finalization of the pivotal trial can lead to changes in the global development plan (10), while later HTA advice may focus on confirming the adequacy of the global development plan, on locally-specific requirements, on PLEG, or a combination of these topics. The correlation between HTA advice and outcome is less clear than for regulatory advice, likely due to confounding factors such as reimbursement (10). While a lack of clear correlation may reduce the influence of HTA advice, it seems logical that compliance with pre-license evidence would have some influence on an HTA assessment, at least on the clinical side. However, considering that many HTA bodies do not have authority over products once they are on the market (9), or prioritize activities related to initial assessment (1), there is a question about the impact of HTA requirements in terms of post-launch evidence development.

### Post-launch evidence generation

LC-HTA approaches may be well suited to encouraging PLEG due to the systematic linkage between different HTA activities. If HTA advice and the initial assessment are clearly connected to a future reassessment, then this creates an incentive for evidence development, especially if the reassessment is connected with reimbursement or access. Systematically linked approaches can work well with individual technology developers who are developing evidence related to addressing technologies with either limited initial evidence or incremental innovation. However, practice changes that impact a technology’s outcomes or changes in health service or delivery context are LC-HTA scenarios where the responsibility of evidence generation may not lie with the technology developer. For such scenarios, HTA bodies may consider (i) collaboration with health providers, technology developers, and academia to monitor for significant new evidence or changes in utilization and (ii) collaboration with health researchers to help set research agendas aligned toward generating evidence relevant to addressing such decision problems (1,7,14).

### Collaboration across jurisdictions

It may be efficient for HTA bodies to collaborate across jurisdictions to define relevant evidence requirements and a common evidentiary database to address core questions, especially in relation to relative effectiveness or rare diseases (1). There are already examples of such collaborations, including the EUnetHTA consortium, the AUS-CAN-UK HTA (15) collaboration, and regional networks in Latin America (16) and Asia Pacific. Standardization of evidence requirements will aid in cross-jurisdictional studies and evidence development planning. Standards and guidelines may be particularly important where the evidence generation is dependent upon emerging methodologies, such as for real-world evidence (RWE). Such guidance is already emerging, for example, the REALISE Working Group (17) and DARWIN-EU (18).

## How to use LC-HTA to inform decision-making across the lifecycle

LC-HTA approaches are especially useful when it is necessary to inform decisions at more than one point in a technology’s LC. In our view, the four scenarios where LC-HTA may be applicable (2) can be grouped into two categories with respect to decision-making:

1. where a decision may have a high risk due to uncertainty related to a limited evidence base, or;
2. where a previous decision may be invalidated due a technology undergoing incremental modification, clinician-led changes in the technology’s utilization, or changes in the health service/delivery context.

### Initial decision uncertainty

In relation to decision-making on the basis of limited or early evidence, LC-HTA approaches have the potential to impact, where applicable, an HTA body’s willingness to tolerate uncertainty. It appears that HTA bodies often use standard review processes for technologies with limited evidence bases resulting in few unrestricted, positive recommendations (12,19). As it is thought that the likelihood of further evidence development and the ability to reassess the initial decision can influence tolerance for uncertainty (4), managed access processes that are linked to evidence generation designed to address the clinical uncertainty have been proposed as a way forwards (5,7,14). The HAS early access authorization program is an example of such a managed access process and features the presumption of added benefit relative to alternatives, the establishment of observational data collection, yearly renewal, and a payback mechanism should the added benefit be lower than initially assumed (20).

### Original decision invalidated

Where the original decision may have become invalidated, a systematic LC-HTA approach can enable efficient determination of whether a decision update is required. At the time of the initial decision, clear parameters could be established. Such parameters would include establishing a process to ‘alert’ the HTA body where there is sufficient change in the technology or its context that a reassessment may be required. An alert system could include establishing criteria to trigger reassessment, establishing evidence collection where change is expected, or collaborating with researchers and health system providers to identify changes in clinical practice or service delivery. A second parameter to follow an alert would be for the HTA body to determine whether a reassessment that results in a change in HTA evaluation would be meaningful for payers, providers, clinicians, or patients. A third parameter would be to focus a reassessment on those aspects of the technology that have changed and avoid duplication of work already completed.

## Effective implementation of LC-HTA into the health ecosystem

To be effectively implemented and impactful, it is crucial to properly integrate the LC-HTA approach into the health ecosystem. There are three key groups of stakeholders that need to be considered: (i) patients and clinicians, (ii) payers and health system decision-makers, and (iii) evidence developers.

### Patients and clinicians

The involvement of patients and clinicians across the entirety of HTA processes is considered essential for HTA bodies (1). In relation to LC-HTA, there are several key areas where patient and clinician involvement would have the greatest potential impact on the approach. The perspectives of patients and clinicians regarding the initial prioritization step, HTA appraisal, and reassessment steps could provide important insights for the HTA body, particularly in relation to the patient’s tolerance for higher risk and to ensure focus on patient needs. Engagement with patients and clinicians might help HTA bodies, payers and providers manage decision risk related to the limitation or withdrawal of technologies following a negative reassessment.

### Payers and health system decision-makers

Payers and health system decision-makers, such as hospital commissioning bodies, are usually the primary recipients of HTA information, which is integrated into their decision-making processes regarding resource allocation. Where there is an unrestricted positive HTA recommendation in the presence of higher uncertainty than standard, then payers and health system decision-makers are taking on additional risk. LC-HTA may help the acceptance of such risk where there is a clear approach aimed at addressing the uncertainty and revisiting the initial recommendation. HTA bodies could partner with these stakeholders to establish managed access processes and providers to embed data collection into their health systems in order to support the LC-HTA approach. For changes to HTA recommendations, especially relating to recommendations for technology withdrawal or limitation, it will be important to have clear and early communication, especially where significant resources have been committed.

### Evidence developers

Evidence development is a key underlying feature of LC-HTA. Where the technology developer is responsible for the evidentiary development, there are multiple engagement touchpoints for the HTA body to consider. The first relates to the clarity of its guidelines and opportunities for dialogue at different points in the LC in order to help ensure that evidence meets the HTA body’s expectations. At the same time, such dialogue can help identify potential issues relating to the feasibility of evidence collection or alerts relating to challenges or delays in evidence collection. The HTA body needs to consider how to incentivize or enforce the technology developer to commit resources to data collection. Where researchers are responsible for evidentiary development, the HTA body needs to identify means by which to focus researchers on the relevant questions for subsequent decision-making (1) and how to ensure such research is undertaken in a timely manner.

## Challenges for LC-HTA approaches

Implementing LC-HTA is not without its challenges, particularly issues relating to resourcing, evidence generation to support subsequent decision-making and decision risk.

### Resourcing and burden of LC-HTA

Resource burden for HTA bodies relevant to LC-HTA has been discussed earlier in this paper and elsewhere (1,5,6,7,21); however, less consideration has been given to resource or burden impacts on other stakeholders. Concern has been raised about the burden on repeated involvement of clinicians and especially patients in an LC-HTA approach (1), hence our recommendation to focus engagement on crucial touchpoints. For technology providers, early scientific dialogue is not always feasible and the extent of evidence development (pre- and post-launch) is linked to commercial considerations, including whether the evidence development is feasible and generalizable (across markets). For providers, it may be practically difficult to respond quickly to changes in HTA decision-making due to the time it takes to procure, supply, and exhaust existing stock (6).

### Evidence generation, privacy, and confidentiality issues

As noted above, LC-HTA approaches may encourage evidence development; however, HTA bodies typically do not have the authority to compel such development, especially in the post-launch space. Potential barriers to evidence development not discussed previously in this paper include technology developer concerns relating to commercially sensitive information (e.g., price) being made public in reassessment reports or academics withholding evidence prior to publication. The challenge of evidence development extends beyond the primary “developer,” be that the technology developer or the researcher, and includes those involved in the provision of the data. Patients may raise privacy concerns, while clinicians and providers may struggle with the administrative burden of data collection (1). Thokla et al. (6) recommend that data sensitivity, copyright, and intellectual property issues should be agreed upon at the outset to ensure alignment with HTA body requirements.

### Methodological dependencies

LC-HTA approaches may have a dependency on the use of RWE, given that the majority of evidence development is expected to occur in the post-launch phase of a technology’s LC. This dependency will relate to the collection, storage, and management of real-world data as well as the statistical transformation, cleaning, and analysis of RWE. Guidelines are required for quality assurance (17) as well as consideration for how to use emerging statistical methodologies. Initiatives, such as the HTx Project, have been developing methods to bridge evidentiary gaps (3). HTA bodies need to consider the implications of such new methodologies for their processes when employing LC_HTA.

### Decision-making and remit

HTA bodies vary in their remit, including whether they are decision-makers or the extent to which they link to downstream decision-making. The extent of remit impacts the ability of HTA bodies to directly impose conditions or even whether they can engage with payers or providers to establish managed access processes or influence methodological guidelines. HTA processes are less impactful if stakeholders such as payers cannot act on them (6), but likewise, the ability to establish LC-HTA processes may be limited if the HTA body is siloed from key stakeholders. Therefore, the extent of remit may therefore dictate the extent to which an HTA body can adopt LC-HTA approaches.

## Conclusion

Considering that HTA bodies operate in the context of their local health and legal systems, with differing levels of resources and remits, the Task Force attempted to identify key considerations common across HTA bodies with respect to building an LC-HTA program. This paper discusses operationalizing the three key steps required to build an LC-HTA approach in order to maximize the approach’s effectiveness and efficiency. We additionally discuss four key factors for consideration when implementing an LC-HTA approach, including both opportunities and challenges. In bringing these steps and factors together, we have developed an operationalization checklist (Table 1) to help HTA bodies develop LC-HTA approaches.

The paper provides two high-level examples of LC-HTA in order to demonstrate the degree of difference that could be expected between LC-HTA approaches optimized towards different decision problems and also to serve as an aid for those considering solutions to these decision problems. Other scenarios see ref (2) may require different sequences, for example, changes in utilization through clinician experience will not lend itself to a discussion on establishing trigger points via early dialogue or technology developer-led evidence development, given that such clinician activity is likely off-label. Likewise, where the health service/delivery context changes, the LC-HTA approach may be more efficient if conducted as a multi-technology reassessment relative to the therapeutic area.

As a next step to advance the discussion about LC-HTA, we believe that the HTA community should consider the development of HTA activity sequencing for the decision problems relating to (i) clinician experimentation and optimization, and (ii) changes in the health service delivery context. A further consideration is how LC-HTA could help or respond to activities of the health system, such as a proactive response to health system needs or in support of de-implementation frameworks (23).
